# Clinical and refractive outcomes of bilateral enhanced monofocal intraocular lens implantation with targeted mini-monovision

**DOI:** 10.1186/s12886-026-05439-8

**Published:** 2026-10-13

**Authors:** Işıl Kefeli Kırcalı, Üzeyir Günenç, Canan Aslı Utine, Gül Arıkan

**Affiliations:** 1 Department of Ophthalmology, Dokuz Eylül University Faculty of Medicine, Mithatpaşa cad No: 1606 Balçova, Izmir, 35340 Turkey; 2 Private Practice, Izmir, Turkey

**Keywords:** Cataract, Extended depth-of-focus (EDOF), Phacoemulsification, Intraocular lens, Lens, RayOne EMV

## Abstract

**Purpose:**

To evaluate visual and refractive outcomes, as well as patient satisfaction after bilateral implantation of an enhanced monofocal intraocular lens (IOL) with targeted mini-monovision.

**Methods:**

We retrospectively reviewed the medical records of patients who underwent bilateral RayOne EMV IOL implantation during cataract surgery with targeted mini-monovision (-0.50 diopters [D] in the non-dominant eye). Patients’ monocular and binocular uncorrected distance visual acuity (UDVA), corrected distance visual acuity (CDVA), uncorrected intermediate visual acuity (UIVA), distance-corrected intermediate visual acuity (DCIVA), and manifest refraction were assessed at the 1st and 6th months postoperatively. Binocular uncorrected and corrected near visual acuities, defocus curves, contrast sensitivity, spectacle independence, dysphotopsia, and patient-reported outcomes were evaluated at Month 6. Patient-reported visual function and satisfaction were assessed using the CatQuest-9SF questionnaire, while spectacle independence and dysphotopsia were evaluated separately using study-specific questions.

**Results:**

Fifty eyes of 25 patients were included. Mean age was 63.64 ± 10.53 years (range: 29–78 years), and the mean follow-up period was 14.52 ± 5.6 months (range: 6–24 months). At 6 months postoperatively, the mean spherical equivalent in dominant eyes was − 0.37 ± 0.29 D (range: -1.00 to + 0.38 D), whereas in non-dominant eyes, it was − 0.78 ± 0.46 D (range: -1.75 to + 0.25 D). The mean binocular UDVA was 0.02 ± 0.03 logMAR (range: 0.00- 0.10 logMAR), while binocular UIVA acuity was 0.21 ± 0.09 logMAR (range: 0.00- 0.40 logMAR) at month 6. Mean binocular uncorrected near visual acuity (UNVA) at 6 months was 0.24 ± 0.12 logMAR, whereas corrected near visual acuity (CNVA) was 0.14 ± 0.08 logMAR (*P* < 0.001). Binocular UDVA was 0.04 logMAR or better in 96% of patients, UIVA was 0.20 logMAR or better in 64%, and uncorrected near visual acuity was 0.3 logMAR or better in 80% of patients. All patients were spectacle independent for distance and intermediate vision; 10/25 patients (40%) were also spectacle independent for near vision, whereas 15/25 (60%) required a near addition. Twenty-four patients (96%) reported being very satisfied with their vision.

**Conclusion:**

Bilateral implantation of an enhanced monofocal IOL with targeted mini-monovision was associated with excellent visual outcomes at intermediate and distance vision with a high level of satisfaction.

## Introduction

Cataract surgery has evolved into a refractive procedure in which postoperative visual quality and spectacle independence have become major determinants of patient satisfaction. Although conventional monofocal intraocular lenses (IOLs) provide excellent distance vision with high optical quality, they offer limited intermediate and near vision, leaving many patients dependent on spectacles for daily activities. As visual demands continue to increase with the widespread use of digital devices, there is growing interest in IOL technologies that extend the range of functional vision while maintaining excellent optical performance. [1, 2]

Diffractive multifocal and extended depth-of-focus (EDOF) IOLs were developed to improve spectacle independence by providing functional vision at multiple distances. While these lenses achieve excellent visual outcomes, light-splitting optics may reduce contrast sensitivity and increase dysphotopsia, including glare and halos, which remain among the most common causes of postoperative dissatisfaction [3–9]. Consequently, preserving optical quality while improving intermediate and functional near vision has become a major focus of contemporary IOL development.

Enhanced monofocal IOLs represent a newer generation of non-diffractive lenses designed to modestly extend the depth of focus while preserving the distance visual acuity and optical quality associated with conventional monofocal lenses [10–12]. Unlike diffractive presbyopia-correcting IOLs, these lenses employ controlled optical modifications that improve intermediate vision while maintaining contrast sensitivity and minimizing photic phenomena. Recent evidence indicates that these lenses improve intermediate and, to a lesser extent, near visual acuity compared with conventional monofocal IOLs, without producing a clinically significant reduction in distance visual acuity [13–15]. The RayOne EMV (Rayner Intraocular Lenses Ltd., Worthing, United Kingdom) is an enhanced monofocal IOL that utilizes controlled positive spherical aberration within the central optic to increase functional depth of focus while preserving the optical characteristics of a conventional monofocal lens [16]. Previous clinical studies have demonstrated excellent distance and intermediate visual acuity, low rates of dysphotopsia, and high patient satisfaction following bilateral implantation of this lens [14, 17–22]. 

Despite the favorable outcomes in the literature, functional near vision remains the principal limitation of enhanced monofocal IOLs when emmetropia is targeted in both eyes. Planned mini-monovision, in which mild residual myopia is intentionally targeted in the non-dominant eye while emmetropia is maintained in the dominant eye, has emerged as an effective strategy to improve near visual performance without sacrificing binocular distance vision or inducing the optical side effects associated with diffractive IOLs [23, 24]. Several studies have demonstrated that low levels of anisometropia improve functional near vision and increase spectacle independence while preserving binocular visual quality and patient satisfaction [25–28]. However, evidence regarding bilateral implantation of the RayOne EMV IOL combined with a mild mini-monovision strategy remains limited.

Although several recent studies have reported favorable outcomes with the RayOne EMV IOL combined with mini-monovision, evidence with a consistently applied − 0.50 D target and concurrent evaluation of the defocus profile, contrast sensitivity, and validated patient-reported outcomes remains limited. Therefore, the present study aimed to evaluate the visual, refractive, and patient-reported outcomes following bilateral implantation of the RayOne EMV enhanced monofocal IOL using a planned mild mini-monovision strategy targeting emmetropia in the dominant eye and approximately − 0.50 D in the non-dominant eye.

## Methods

This retrospective observational study included consecutive patients who underwent bilateral cataract surgery with implantation of the RayOne EMV enhanced monofocal intraocular lens (Rayner Intraocular Lenses Ltd., Worthing, UK) at the Department of Ophthalmology, Dokuz Eylül University Faculty of Medicine. The medical records of 25 patients who underwent this procedure between December 2022 and April 2024 were screened. During the study period, 25 consecutive patients underwent bilateral RayOne EMV implantation with the planned mini-monovision protocol. All 25 met the eligibility criteria, none were excluded or lost to follow-up, and complete Month 6 outcome data were available for all patients. Therefore, 25 patients (50 eyes) were included in the final analysis.

The study adhered to the tenets of the Declaration of Helsinki and was approved by the Institutional Ethics Committee of Dokuz Eylül University (Approval No. 2024/28 − 11). Written informed consent was obtained from all participants before surgery. Patients were eligible for inclusion if they had undergone bilateral cataract surgery with implantation of the RayOne EMV IOL, had a calculated IOL power between + 10.0 and + 30.0 D, preoperative corneal astigmatism ≤ 1.00 D, and no ocular pathology other than cataract. The axial length of the included eyes ranged from 21.74 to 24.54 mm; therefore, no eyes with extreme axial lengths were present in the study. Eyes with corneal astigmatism higher than 1.00 D, corneal dystrophy, pseudoexfoliation syndrome, zonular weakness, retinal diseases such as age-related macular degeneration or diabetic retinopathy; and any history of previous ocular surgery were excluded.

### Preoperative assessment

All patients underwent a comprehensive ophthalmic examination including manifest refraction, keratometry, slit-lamp biomicroscopy, Goldmann applanation tonometry, dilated fundus examination, optical coherence tomography, and optical biometry using the IOLMaster (Carl Zeiss Meditec, Jena, Germany). For each patient, the appropriate IOL power was calculated using the SRK/T formula. Ocular dominance was determined using the hole-in-the-card test. For the dominant eye, the IOL power corresponding most closely to emmetropia was selected. For the non-dominant eye, the IOL power corresponding most closely to a predicted postoperative spherical equivalent of approximately − 0.50 D was selected.

A mild mini-monovision strategy was planned for all patients, targeting emmetropia in the dominant eye and approximately − 0.50 D in the non-dominant eye.

### Intraocular lens

The RayOne EMV RAO200E (Rayner Intraocular Lenses Ltd., Worthing, UK) is a single-piece hydrophilic acrylic enhanced monofocal intraocular lens with a 6.0-mm aspheric optic, an overall diameter of 12.5 mm, a water content of 26%, and a preloaded injector system. Its patented optical design uses controlled positive spherical aberration to extend the depth of focus while minimizing light loss and preserving contrast sensitivity, providing approximately 1.50 D of functional depth of focus. These features aim to improve intermediate and functional near vision while maintaining excellent distance visual acuity and a low incidence of photic phenomena [16]. 

### Surgical technique

All surgeries were performed by a single experienced surgeon (U.G.) under topical anesthesia using a standardized phacoemulsification technique. The eye with poorer visual acuity was operated on first, followed by surgery on the fellow eye approximately 1 week later.

A 2.4-mm clear corneal incision and an approximately 5.5-mm continuous curvilinear capsulorhexis were created. Following phacoemulsification with the stop-and-chop technique and cortical aspiration, the RayOne EMV IOL was implanted in the capsular bag using the preloaded injector system. At the end of surgery, the ophthalmic viscosurgical device was completely removed, stromal hydration was performed, and intracameral cefuroxime (1.0 mg/0.1 mL) was administered.

### Postoperative assessment

Postoperative examinations were performed on day 1, week 1, and months 1 and 6. Monocular and binocular uncorrected distance visual acuity (UDVA), corrected distance visual acuity (CDVA), binocular uncorrected intermediate visual acuity (UIVA) and distance-corrected intermediate visual acuity (DCIVA) at 66 cm were assessed at postoperative Months 1 and 6. Binocular uncorrected near visual acuity (UNVA) and corrected near visual acuity (CNVA) at 40 cm were assessed at the postoperative Month 6 visit.

Manifest refraction, spherical equivalent, slit-lamp biomicroscopy, intraocular pressure, and photopic contrast sensitivity were also recorded. Contrast sensitivity was measured binocularly with the Mars Letter Contrast Sensitivity Test (Mars Perceptrix, Chappaqua, NY, USA) at a viewing distance of 50 cm under photopic room illumination (chart luminance approximately 85 cd/m²), with the distance correction plus a + 2.00 D near addition in place; letters were scored individually (0.04 log units per letter) according to the manufacturer’s instructions. Results were compared with the age-related normative limits reported by Haymes et al. [29]

The defocus curve was obtained binocularly at the Month 6 visit with the full manifest distance refraction in place in both eyes under photopic conditions (85 cd/m^2^), with defocus values between + 2.00 D and − 4.00 D in 0.50 D steps.

Patient-reported visual function and satisfaction were evaluated using the Catquest-9SF questionnaire, a previously published and validated nine-item patient-reported outcome measure designed to assess vision-related difficulties in activities of daily living and satisfaction with vision. The original development and validation study of the Catquest-9SF has been previously published and is cited accordingly [30]. Spectacle independence and dysphotopsia (glare and halo) were assessed separately using additional study-specific questions administered at the same visit.

### Statistical analysis

Statistical analyses were performed with SPSS Statistics 26.0 (IBM Corp., Armonk, NY, USA). As most variables were non-normally distributed according to the Shapiro–Wilk test, continuous data are presented as mean ± SD and median (IQR), and visual acuity was analyzed in logMAR. Binocular outcomes (patient level, *n* = 25) were compared between Months 1 and 6 using the Wilcoxon signed-rank test. Monocular longitudinal outcomes (50 eyes of 25 patients) were analyzed using linear mixed-effects models with visit as a fixed effect and random effects for patient and for eye nested within patient to account for repeated measurements and inter-eye correlation. Dominant-versus-non-dominant eye comparisons and UNVA-versus-CNVA comparisons were analyzed using the Wilcoxon signed-rank test. For paired patient-level comparisons, mean paired differences with 95% confidence intervals were additionally reported descriptively to quantify the magnitude and uncertainty of change. P values were adjusted using the Holm procedure within families of related comparisons (binocular visits, monocular visits, and eye dominance), and an adjusted *P* < 0.05 was considered statistically significant. These analyses were added during revision and should be regarded as exploratory.

## Results

### Patient characteristics

Fifty eyes of 25 patients who underwent bilateral RayOne EMV implantation were included. During the study period, 25 consecutive patients underwent bilateral RayOne EMV implantation with the planned mini-monovision protocol. All met the eligibility criteria, no patient was excluded, and none was lost to follow-up; all 25 patients (50 eyes) attended the Month 6 visit with complete outcome data and were included in the analysis. Patients’ demographics and preoperative characteristics are summarized in Table 1.

**Table 1:** Patients’ preoperative characteristics

Characteristic	Value
Female, n (%)	12 (48)
Male, n (%)	13 (52)
Age (years), mean ± SD	63.64 ± 10.53
Axial length (mm), mean ± SD	23.20 ± 0.69
Mean keratometry (D), mean ± SD	43.50 ± 1.53
Corneal astigmatism (D), mean ± SD	0.57 ± 0.37
IOL power (D), mean ± SD	21.65 ± 2.89
Dominant eye	
Right, n (%)	13 (52)
Left, n (%)	12 (48)

SD =standard deviation

### Refractive outcomes

The mean postoperative spherical equivalent changed from − 0.68 ± 0.41 D (median − 0.75, IQR − 0.75 to − 0.50) at Month 1 to − 0.57 ± 0.44 D (median − 0.50, IQR − 0.75 to − 0.25) at Month 6 (mixed-model mean change + 0.11 D, 95% CI 0.03 to 0.18; *P* = 0.008, Holm-adjusted *P* = 0.017), i.e., less than the 0.25 D step of manifest refraction. At 6 months, when refractive accuracy was evaluated relative to the nominal protocol targets (plano for the dominant eye and − 0.50 D for the non-dominant eye), 39 of 50 eyes (78%) were within ± 0.50 D and 49 of 50 eyes (98%) were within ± 1.00 D of the intended refractive target. In dominant eyes, for which the nominal target was plano, the signed deviation from target is numerically identical to the achieved spherical equivalent (− 0.37 ± 0.29 D), with a mean absolute deviation from target of 0.40 D; 84% and 100% of eyes were within ± 0.50 D and ± 1.00 D, respectively. In non-dominant eyes, the achieved spherical equivalent of − 0.78 ± 0.46 D corresponds to a mean deviation from the − 0.50 D target of − 0.28 ± 0.46 D, and the mean absolute deviation was 0.41 D, with 72% and 96% of eyes within ± 0.50 D and ± 1.00 D, respectively (Table 2).

Mean postoperative refractive astigmatism at 6 months was − 0.75 ± 0.43 D.

Consistent with the planned mini-monovision strategy, the dominant eyes achieved a mean spherical equivalent of − 0.37 ± 0.29 D, whereas the non-dominant eyes demonstrated a significantly more myopic refraction (− 0.78 ± 0.46 D; mean paired difference − 0.41 D, 95% CI − 0.56 to − 0.25; Holm-adjusted *P* < 0.001). Mean monocular UDVA was numerically better in dominant eyes than in non-dominant eyes (0.06 ± 0.07 vs. 0.09 ± 0.09 logMAR); however, this difference was not statistically significant (mean paired difference 0.03 logMAR, 95% CI − 0.003 to 0.07; Holm-adjusted *P* = 0.107).

The achieved interocular refractive difference (dominant minus non-dominant eye spherical equivalent) was 0.41 ± 0.38 D (median 0.25 D, IQR 0.13–0.63 D; range 0.00–1.38 D) demonstrating interindividual variability in the achieved degree of mini-monovision.

**Table 2:** Refractive outcomes and monocular UDVA at Month 6 by ocular dominance (*n* = 25 patients, 50 eyes)

Parameter	Dominant eyes (target plano)	Non-dominant eyes (target − 0.50 D)	Paired difference, ND − D (95% CI)	Holm-adjusted *P*
Spherical equivalent (D), mean ± SD	−0.37 ± 0.29	−0.78 ± 0.46	−0.41 (− 0.56 to − 0.25)	< 0.001
Spherical equivalent (D), median (IQR)	−0.38 (− 0.50 to − 0.25)	−0.75 (− 1.00 to − 0.50)		
Spherical equivalent (D), range	−1.00 to + 0.38	−1.75 to + 0.25		
Signed deviation from nominal target (D), mean ± SD	−0.37 ± 0.29	−0.28 ± 0.46		
Mean absolute deviation from target (D)	0.40	0.41		
Within ± 0.50 D of target, n (%)	21 (84)	18 (72)		
Within ± 1.00 D of target, n (%)	25 (100)	24 (96)		
Monocular UDVA (logMAR), mean ± SD	0.06 ± 0.07	0.09 ± 0.09	0.03 (− 0.003 to 0.07)	0.107
Monocular UDVA (logMAR), median (IQR)	0.00 (0.00–0.10)	0.10 (0.04–0.10)		

**Achieved interocular difference (dominant − non-dominant SE)**,** per patient**: 0.41 ± 0.38 D; median 0.25 D (IQR 0.13–0.63); range 0.00–1.38 D. Fifteen patients (60%) achieved an interocular difference within ± 0.25 D of the intended 0.50-D target, whereas 4 patients (16%) had no interocular refractive difference

D = dominant; ND = non-dominant; SE = spherical equivalent; UDVA = uncorrected distance visual acuity; IQR = interquartile range; CI = confidence interval. Deviation from target = achieved SE minus nominal protocol target; these values are not IOL formula prediction errors. Paired comparisons: Wilcoxon signed-rank test (*n* = 25 pairs); 95% CI of the mean paired difference (t distribution). Holm adjustment within the dominance family (2 tests)

### Visual acuity

Postoperative visual acuity outcomes are summarized in Table 3.

Mean monocular UDVA improved from 0.12 ± 0.08 logMAR at Month 1 to 0.07 ± 0.08 logMAR at Month 6 (mixed-model mean change − 0.04 logMAR, 95% CI − 0.05 to − 0.03, approximately two letters; Holm-adjusted *P* < 0.001), whereas monocular CDVA was at or near ceiling at both visits (0.00 ± 0.02 and 0.00 ± 0.01 logMAR; Holm-adjusted *P* = 0.077), having improved from 0.43 ± 0.13 logMAR preoperatively (Holm-adjusted *P* < 0.001). Binocular UDVA remained stable throughout follow-up (0.02 ± 0.03 logMAR at both visits), as did binocular CDVA (0.01 ± 0.02 logMAR) (Holm-adjusted *P* = 0.250 for both).

Binocular UIVA was 0.22 ± 0.10 logMAR at Month 1 and 0.21 ± 0.09 logMAR at Month 6; however, this difference was not statistically significant (mean change − 0.004 logMAR, 95% CI − 0.018 to 0.010; *P* = 0.414, Holm-adjusted *P* = 0.414). Binocular DCIVA changed from 0.18 ± 0.07 logMAR at Month 1 to 0.17 ± 0.07 logMAR at Month 6 (mean change − 0.018 logMAR, 95% CI − 0.033 to − 0.002; unadjusted *P* = 0.039, Holm-adjusted *P* = 0.157). This change corresponds to less than one letter (0.02 logMAR per letter), is well below the one-line (0.10 logMAR) change generally regarded as clinically meaningful, and lies within the test–retest variability of chart-based acuity measurement; it is therefore not considered clinically relevant.

At 6 months, 96% of patients achieved binocular UDVA of 0.04 logMAR or better, and all patients achieved 0.10 logMAR or better. Monocular UDVA was 0.10 logMAR or better in 78% of eyes and 0.30 logMAR or better in all eyes. Binocular UIVA was 0.20 logMAR or better in 16 patients (64%).

Mean binocular uncorrected near visual acuity (UNVA) at 6 months was 0.24 ± 0.12 logMAR (median 0.20, IQR 0.20–0.30), whereas corrected near visual acuity (CNVA) was 0.14 ± 0.08 logMAR (median 0.15, IQR 0.10–0.20; mean difference 0.10 logMAR, 95% CI 0.06–0.14; *P* < 0.001). 80% of patients achieved binocular uncorrected near visual acuity of 0.30 logMAR or better.

**Table 3:** Visual acuity (logMAR) and spherical equivalent at postoperative Months 1 and 6

Outcome	*n*	Month 1	Month 6	Mean change (95% CI)	*P*	Holm-adjusted *P*
*Binocular (patient level)*						
UDVA	25	0.02 ± 0.03 0.00 (0.00–0.04)	0.02 ± 0.03 0.00 (0.00–0.04)	−0.006 (− 0.012 to 0.001)	0.102	0.250
CDVA	25	0.01 ± 0.02 0.00 (0.00–0.04)	0.01 ± 0.02 0.00 (0.00–0.00)	−0.005 (− 0.010 to 0.001)	0.083	0.250
UIVA	25	0.22 ± 0.10 0.20 (0.20–0.30)	0.21 ± 0.09 0.20 (0.20–0.30)	−0.004 (− 0.018 to 0.010)	0.414	0.414
DCIVA	25	0.18 ± 0.07 0.20 (0.10–0.20)	0.17 ± 0.07 0.20 (0.10–0.20)	−0.018 (− 0.033 to − 0.002)	0.039	0.157
UNVA	25	NA	0.24 ± 0.12 0.20 (0.20–0.30)			
CNVA	25	NA	0.14 ± 0.08 0.15 (0.10–0.20)	CNVA − UNVA: −0.10 (− 0.14 to − 0.06)	< 0.001	NA (single test)
*Monocular (eye level; 50 eyes of 25 patients)*						
UDVA	50	0.12 ± 0.08 0.10 (0.04–0.15)	0.07 ± 0.08 0.04 (0.00–0.10)	−0.042 (− 0.054 to − 0.030)^a^	< 0.001^a^	< 0.001
CDVA	50	0.00 ± 0.02 0.00 (0.00–0.00)	0.00 ± 0.01 0.00 (0.00–0.00)	−0.002 (− 0.005 to 0.000)^a^	0.077^a^	0.077
SE (D)	50	−0.68 ± 0.41 −0.75 (− 0.75 to − 0.50)	−0.57 ± 0.44 −0.50 (− 0.75 to − 0.25)	+ 0.11 (0.03 to 0.18)^a^	0.008^a^	0.017

Values are mean ± SD (upper line) and median (IQR) (lower line). Binocular outcomes are patient-level measurements (*n* = 25 patients); monocular outcomes are eye-level measurements (*n* = 50 eyes of 25 patients). Negative change indicates improvement in logMAR acuity; positive change in SE indicates a hyperopic shift. Binocular comparisons and the CNVA–UNVA comparison: Wilcoxon signed-rank test; mean paired difference with 95% CI (t distribution). ^a^Linear mixed-effects model with a random intercept for patient and a variance component for eye nested within patient; estimate, 95% CI, and P value from the model. Sensitivity analysis with both eyes averaged per patient (Wilcoxon signed-rank, *n* = 25): UDVA *P* < 0.001, CDVA *P* = 0.083, SE *P* = 0.015. Holm adjustment was applied within the binocular family (4 tests) and the monocular family (4 tests, including preoperative versus Month 6 CDVA). Preoperative monocular CDVA was 0.43 ± 0.13 logMAR [median 0.40 (0.30–0.50)]; model-estimated change to Month 6 − 0.43 (95% CI − 0.46 to − 0.40), Holm-adjusted *P* < 0.001. UNVA and CNVA were measured only at Month 6; NA = not available. All inferential analyses in this table were added during peer review and are exploratory. UDVA = uncorrected distance visual acuity, CDVA = corrected distance visual acuity, UIVA = uncorrected intermediate visual acuity, DCIVA = distance-corrected intermediate visual acuity, UNVA = uncorrected near visual acuity, CNVA = corrected near visual acuity, SE = spherical equivalent, IQR = interquartile range, CI = confidence interval

### Defocus curve and contrast sensitivity

**Fig. 1:**
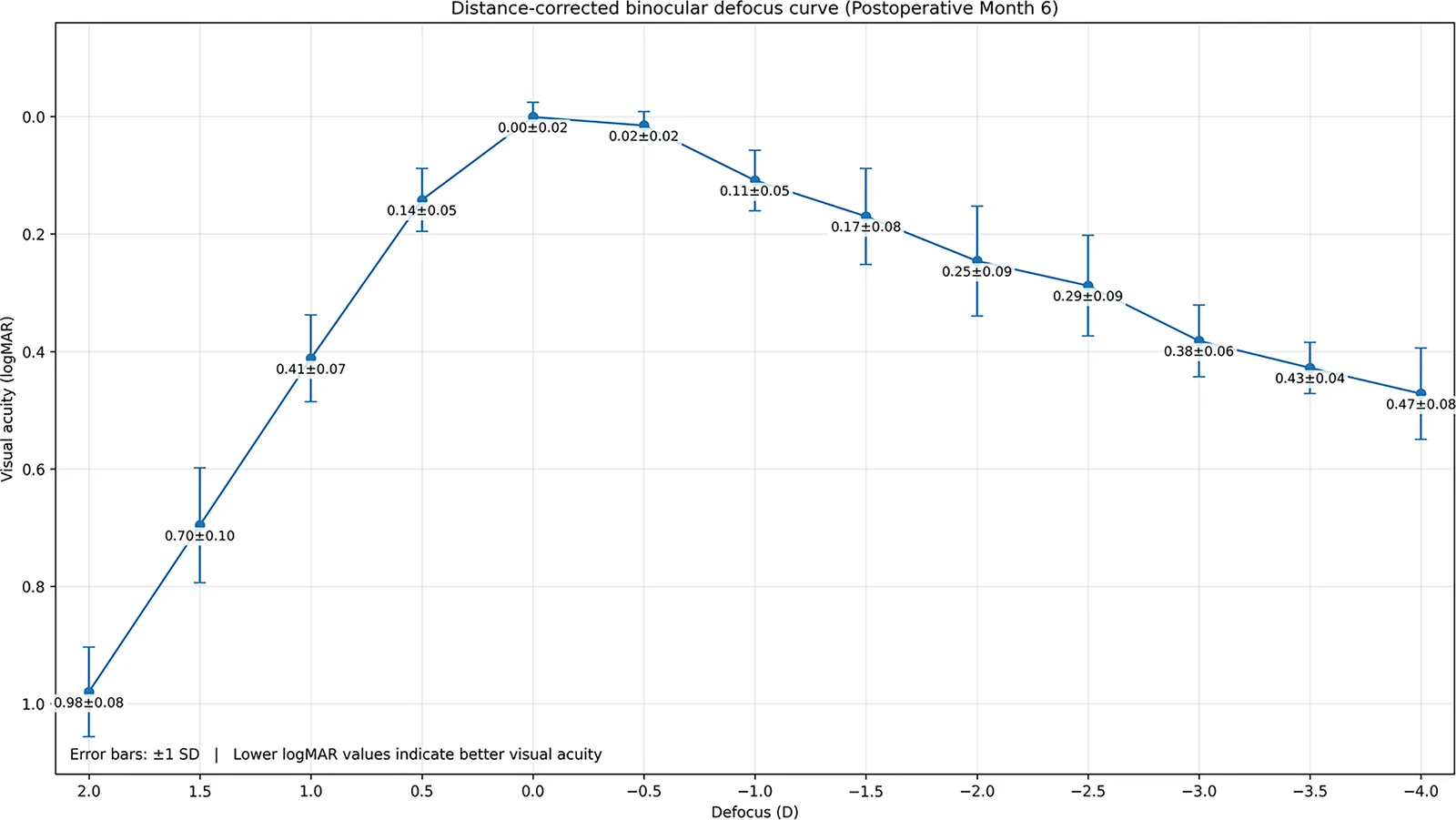
Distance-corrected binocular defocus curve at postoperative Month 6. Values are presented as mean logMAR visual acuity ± standard deviation (SD); error bars represent ± 1 SD. All 25 patients contributed measurements at each tested defocus level. Lower logMAR values indicate better visual acuity

The binocular defocus curve demonstrated a peak at plano with a gradual decline in visual acuity toward negative defocus levels. At − 1.50 D, corresponding approximately to a viewing distance of 66 cm, mean binocular visual acuity on the defocus curve was 0.17 ± 0.08 logMAR (*n* = 25) (Fig. 1).

Mean binocular photopic contrast sensitivity at 6 months was 1.61 ± 0.13 log units. For reference, the age-related monocular 95% normative limits reported for the Mars test at 60 years of age are approximately 1.52–1.68 log units [29]. 

### Spectacle independence and patient satisfaction

Complete spectacle independence for distance and intermediate vision was achieved in all patients. Ten patients (40%) remained spectacle independent for near vision, whereas 15 (60%) required a near addition for reading at 40 cm or closer. The mean near addition was 1.33 ± 0.34 D.

Responses to the Catquest-9SF questionnaire indicated a high level of patient satisfaction. Twenty-four patients (96%) reported being very satisfied with their vision, while one patient (4%) was fairly satisfied. Overall, 96% reported no visual difficulties in daily life, and between 88% and 100% reported no difficulty performing most daily visual tasks.

At 6 months, no glare or halo symptoms were reported by 80% of patients. Mild symptoms were reported by 12%, and moderate symptoms by 8%; however, none considered these symptoms bothersome in daily life.

### Complications

Posterior capsule opacification developed in 4 eyes of 3 patients. The mean interval between surgery and posterior capsule opacification development was 10 ± 1.8 months (range: 8–12 months). All patients with posterior capsule opacification were successfully treated with Nd: YAG laser capsulotomy. No intraoperative complications or posterior segment complications occurred during follow-up.

## Discussion

The present study evaluated the visual and refractive outcomes of bilateral RayOne EMV implantation combined with a mild mini-monovision strategy. The non-dominant eye was targeted for approximately − 0.50 D of myopia. After this combined approach, patients had excellent binocular distance vision, good intermediate vision, and functional near vision; all patients were spectacle independent for distance and intermediate tasks, whereas 60% still required a near addition. These favorable outcomes should nonetheless be interpreted in the context of the relatively small sample size of this single-center series (25 patients, 50 eyes).

In the present study, 6 months after the surgery, mean monocular and binocular UDVA were 0.07 ± 0.08 and 0.02 ± 0.03 logMAR, respectively. Mean binocular UIVA was 0.22 ± 0.10 logMAR and 0.21 ± 0.09 logMAR at months 1 and 6, respectively. All patients achieved complete spectacle independence for distance and intermediate vision. Intermediate acuity was stable between Months 1 and 6; the 0.02 logMAR change in DCIVA was not statistically significant after adjustment for multiple comparisons and is below the threshold of clinical relevance. These findings are consistent with good distance and intermediate visual performance and high patient satisfaction after bilateral RayOne EMV implantation with mild mini-monovision.

Binocular UDVA was also better than monocular UDVA (0.02 vs. 0.07 logMAR at Month 6). This is expected from binocular summation and from the fact that binocular distance acuity is largely determined by the better-seeing, distance-targeted eye; in the absence of a control group, this difference cannot be attributed to an increase in depth of focus produced by the IOL design or the mini-monovision strategy.

A recent systematic review and meta-analysis reported that enhanced monofocal IOLs significantly improved intermediate and near visual acuity compared with conventional monofocal IOLs, while maintaining comparable distance visual acuity, contrast sensitivity, and rates of photic phenomena [24]. The excellent binocular distance acuity and good intermediate performance observed in the present study are consistent with this expected clinical profile.

Our findings are also consistent with previous studies evaluating the RayOne EMV IOL. Madhivanan et al. [18] reported a mean binocular UDVA of 0.00 ± 0.09 logMAR following bilateral RayOne EMV implantation, demonstrating excellent distance visual outcomes. Similarly, García-Bella et al. [19] reported a mean binocular UDVA of 0.01 ± 0.08 logMAR and a binocular uncorrected intermediate visual acuity of 0.13 ± 0.07 logMAR after bilateral RayOne EMV implantation with targeted emmetropia, confirming excellent distance and intermediate visual performance.

Useful near visual performance was observed after the combined implantation and refractive-targeting strategy. In our study, the mean binocular uncorrected near visual acuity (UNVA) reached 0.24 ± 0.12 logMAR (range: 0.0 to 0.4 logMAR), which further improved to a mean binocular corrected near visual acuity (CNVA) of 0.14 ± 0.08 logMAR (range: 0.0 to 0.3 logMAR) (mean difference 0.10 logMAR, 95% CI 0.06–0.14; *P* < 0.001). This statistically significant difference indicates that despite functional uncorrected performance, near vision was further optimized with the aid of a near correction. This near performance was observed together with the planned refractive offset; at Month 6, the mean spherical equivalent was − 0.37 ± 0.29 D in dominant eyes and − 0.78 ± 0.46 D in non-dominant eyes, and 80% of patients achieved a binocular UNVA of 0.30 logMAR or better. Because there was no control group, the separate contributions of the IOL optics and of the myopic offset in the non-dominant eye to this outcome cannot be determined. Only 40% of patients were spectacle independent for near vision, and 60% of patients still required a low near addition of 1.33 ± 0.34 D for prolonged reading and other demanding near activities.

Overall, the outcomes observed after combining the RayOne EMV IOL with mild mini-monovision suggest a favorable balance between excellent distance and intermediate vision and functional, daily-life near visual performance.

Law et al. [21] evaluated bilateral RayOne EMV implantation combined with a mild mini-monovision strategy and reported mean binocular uncorrected distance, intermediate, and near visual acuities of 0.05 ± 0.07, 0.16 ± 0.11, and 0.30 ± 0.17 logMAR, respectively. These results are comparable with our outcomes and are consistent with the previous reports of excellent distance and intermediate vision together with functional near vision.

Our results were also characterized by a low incidence of bothersome photic phenomena, and high patient satisfaction. The intentional myopic target in the non-dominant eye was achieved without a clinically relevant compromise in binocular distance visual acuity, which is compatible with the view that mild mini-monovision can be combined with the RayOne EMV without a clinically relevant loss of binocular distance visual acuity [25, 26, 28]. 

The refractive results also demonstrated reasonable predictability. At 6 months, when refractive accuracy was evaluated relative to the nominal protocol targets, 78% of eyes were within ± 0.50 D and 98% were within ± 1.00 D of their respective targets. The mean absolute deviation from target was 0.40 D in dominant eyes and 0.41 D in non-dominant eyes. Accurate refractive targeting remains particularly important with mini-monovision because an unintended hyperopic outcome in the dominant eye or excessive myopia in the non-dominant eye may reduce binocular performance. In this study, the mean spherical equivalent was − 0.37 D in dominant eyes and − 0.78 D in non-dominant eyes. Both groups were slightly more myopic than their nominal targets. The mean achieved interocular difference was 0.41 ± 0.38 D (median 0.25 D), smaller than the intended 0.50 D; only 60% of patients were within ± 0.25 D of the intended difference, and no interocular difference was achieved in 4 patients (16%). The intended interocular difference was therefore only partially achieved at the individual level. These values describe deviation from the nominal protocol targets and should not be interpreted as IOL formula prediction error.

A key feature of the present study was the use of mild mini-monovision. Targeting the non-dominant eye for slight myopia can extend functional vision toward near distances without producing the degree of anisometropia associated with conventional monovision. In the current study, non-dominant eyes had significantly more myopic refractive outcomes than dominant eyes, whereas the difference in monocular UDVA (0.03 logMAR) was small and not statistically significant. However, binocular uncorrected distance acuity remained excellent, consistent with good binocular tolerance of the induced refractive difference. This finding is compatible with, but does not demonstrate, the concept that the moderate extension of focus provided by an enhanced monofocal IOL may compensate for the reduction in monocular distance vision caused by mild myopia in the non-dominant eye [28]. 

Similar benefits have been reported with other enhanced monofocal and non-diffractive EDOF lenses. Park et al. [31] found that mini-monovision with the Tecnis Eyhance IOL improved near vision and spectacle independence without compromising distance and intermediate visual acuity or causing a meaningful deterioration in contrast sensitivity. A comparative study by Solomon et al. [25] showed that targeting the non-dominant eye for slight myopia using the Vivity IOL improved intermediate and near performance while preserving excellent binocular distance vision. In that study, contrast sensitivity and visual disturbances were comparable to those obtained with a conventional monofocal IOL. These observations are consistent with the present findings and suggest that mild mini-monovision can extend the functional range of enhanced monofocal and EDOF IOLs without sacrificing their principal optical advantages.

Because this retrospective series lacked an internal or external control group, the specific contribution of the RayOne EMV optical design cannot be fully separated from the effect of the mini-monovision strategy itself. Previous studies have shown that pseudophakic mini-monovision with conventional monofocal IOLs can improve functional intermediate and near vision and reduce spectacle dependence by intentionally inducing mild myopia in the non-dominant eye [13, 15]. However, enhanced monofocal IOLs may offer an additional advantage by extending the depth of focus through their optical design, potentially providing greater intermediate visual performance with a relatively small degree of anisometropia. Comparative evidence across monofocal, enhanced monofocal, and EDOF platforms suggests that both IOL design and refractive targeting contribute to the final binocular range of vision [26, 27]. In this context, the favorable intermediate and functional near outcomes observed in the present study likely reflect a combined effect of the RayOne EMV optical profile and the planned − 0.50 D mini-monovision strategy rather than either component in isolation. Nevertheless, direct head-to-head comparisons between bilateral RayOne EMV mini-monovision and conventional monofocal mini-monovision remain limited. Mixed-IOL strategies represent another approach to extending the binocular range of vision by combining lenses with different optical characteristics between the two eyes [8, 9]. For example, Brar et al. [32] reported favorable visual and refractive outcomes and high patient satisfaction following implantation of a conventional monofocal IOL in one eye and an extended-range-of-vision IOL in the contralateral eye with mini-monovision, supporting the feasibility of such mixed-IOL strategies. In contrast, bilateral implantation of the same enhanced monofocal IOL, as performed in the present study, maintains the same IOL platform in both eyes while using a modest interocular refractive difference to extend functional vision. Whether this approach provides advantages in binocular summation, stereopsis, visual quality, or patient tolerance compared with conventional monofocal mini-monovision or mixed-IOL strategies cannot be determined from the present study. Prospective randomized studies directly comparing these strategies are therefore warranted to determine the relative contributions of IOL optical design and refractive targeting to visual performance, spectacle independence, and patient-reported outcomes.

Near vision remains the main limitation of enhanced monofocal lenses compared with trifocal IOLs. In the present study, the mean uncorrected near binocular visual acuity at 40 cm was 0.24 logMAR, and 80% of patients achieved 0.30 logMAR or better. Nevertheless, only 40% of patients were completely spectacle independent for near activities, while 60% required an addition for sustained reading or fine work at 40 cm or closer. These results demonstrate an important distinction between measurable functional near acuity and complete near spectacle independence. The − 0.50 D target provided useful near vision for many daily activities but was not sufficient to eliminate the need for reading glasses in most patients. A greater myopic target could potentially improve near spectacle independence, but this benefit must be balanced against possible reductions in monocular distance acuity, stereopsis, and tolerance of anisometropia. Can et al. [14] evaluated bilateral RayOne EMV implantation using a mild mini-monovision strategy targeting − 0.70 D of myopia in the non-dominant eye. They reported mean binocular uncorrected distance, intermediate, and near visual acuities of 0.00 ± 0.03, 0.00 ± 0.02, and 0.01 ± 0.02 logMAR, respectively. Notably, visual outcomes were comparable to those achieved with the PanOptix trifocal IOL, while demonstrating a lower incidence of dysphotopsia and better contrast sensitivity.

The binocular defocus curve further supports the clinical visual acuity findings. Visual acuity peaked around plano and declined gradually with increasing negative defocus, rather than demonstrating clearly separated focal points. At − 1.50 D, corresponding approximately to a viewing distance of 66 cm, mean binocular visual acuity was 0.17 logMAR. This continuous decline is compatible with an extended-range rather than a multifocal optical profile. Recent clinical and aberrometric evaluations of the RayOne EMV have similarly demonstrated maintained distance acuity with improved functional intermediate vision and an extended range of focus of approximately 1.3–1.5 D [17, 33, 34]. Because the defocus curve was measured with full manifest distance correction in both eyes, the planned interocular refractive offset was neutralized during testing; the curve therefore reflects the binocular defocus tolerance of the implanted IOLs and does not provide evidence of the effect of the mini-monovision strategy.

Visual quality is particularly relevant when considering an enhanced monofocal IOL as an alternative to diffractive multifocal lenses. Mean binocular photopic contrast sensitivity in the present study was 1.61 log units which is comparable to the age-related monocular normative limits reported for this test [29]; however, binocular values are expected to exceed monocular values owing to binocular summation, and in the absence of preoperative or control measurements, this finding does not demonstrate normal or preserved contrast sensitivity. In addition, 80% of patients reported no glare or halo, while the remaining patients reported only mild or moderate symptoms. None considered these phenomena sufficiently disturbing to interfere with everyday activities. These findings are consistent with the optical principle underlying enhanced monofocal lenses, which extend the range of focus without splitting incoming light among several discrete focal points.

Patient-reported outcomes were consistent with the objective visual results. Almost all patients reported no meaningful visual difficulty in daily life. According to the CatQuest-9SF questionnaire, 96% of patients were very satisfied with their postoperative vision. Difficulties were most frequently reported for reading newspaper text, which agrees with the finding that a majority of patients continued to require an addition for demanding near tasks. By contrast, most patients reported no difficulty with face recognition, shopping, walking on uneven surfaces, reading television subtitles, or performing hobbies. This pattern suggests that RayOne EMV with mild mini-monovision was associated with a broad range of functional vision suitable for most daily activities. At the same time, patients should still be counseled that spectacles may be necessary for prolonged reading and fine near work. In a recent RayOne EMV monovision study, Llovet-Rausell et al. [22] similarly reported high satisfaction, preserved contrast sensitivity, and good functional vision across distance and intermediate ranges.

No posterior segment complications or other serious adverse events occurred. Posterior capsule opacification requiring Nd: YAG laser capsulotomy developed in four eyes of three patients.

The mean clinical follow-up duration in the present study was 14.5 months; however, the standardized visual, refractive, defocus-curve, contrast-sensitivity, and patient-reported outcome assessments analyzed in this study were performed through postoperative Month 6. Intermediate visual acuity did not change meaningfully between Months 1 and 6. To our knowledge, this is also among the first reports to combine a consistently applied − 0.50 D mini-monovision target with concurrent evaluation of the binocular defocus curve, photopic contrast sensitivity, and a validated patient-reported outcome measure (CatQuest-9SF) in the same study of RayOne EMV recipients, clarifying how this lens performs across multiple complementary outcome domains rather than in isolation.

This study has several limitations. First, the relatively small sample size of this series (25 patients, 50 eyes), together with its retrospective and single-center design, limits the statistical power and generalizability of the findings. Second, the contribution of the RayOne EMV optical design cannot be completely separated from the effect of mini-monovision. Contrast sensitivity was assessed only under photopic conditions, and mesopic contrast sensitivity with and without glare would have provided a more comprehensive evaluation of visual quality. The mixed-effects models, confidence intervals, and multiplicity adjustment were added during peer review and were not prespecified; all inferential results should therefore be regarded as exploratory. Refractive accuracy was assessed against nominal protocol targets rather than the eye-specific predicted refraction, so formula prediction error could not be evaluated. The defocus curve was obtained with full distance correction in both eyes and therefore does not capture the effect of the mini-monovision strategy. Contrast sensitivity was not measured preoperatively. Finally, the study included only patients without clinically relevant ocular comorbidity, and the findings may therefore not be directly applicable to a broader cataract population. Although eye-level outcomes were analyzed with mixed-effects models that account for inter-eye correlation and repeated measurements, the small number of patients limits the precision of these estimates.

Despite these limitations, the study has several strengths. All procedures were performed by a single experienced surgeon using a standardized surgical technique, and all patients underwent bilateral implantation with the same IOL and a consistent mini-monovision protocol. The evaluation included monocular and binocular visual acuity at multiple distances, refractive outcomes, a binocular defocus curve, contrast sensitivity, spectacle independence, dysphotopsia, and a validated patient-reported outcome measure. The follow-up period was also longer than the 1- to 3-month follow-up used in many previous studies, allowing assessment after refractive stabilization and neuroadaptation.

In conclusion, bilateral implantation of the RayOne EMV enhanced monofocal IOL combined with mild mini-monovision was associated with excellent distance vision, good intermediate vision, and useful functional near vision. All patients were spectacle independent for distance and intermediate activities and 40% also for near, whereas 60% required reading glasses for demanding near tasks. A low incidence of bothersome dysphotopsia, and high patient satisfaction suggest that this combined approach may represent an effective option for patients seeking a broader range of vision while wishing to avoid the visual disturbances associated with diffractive multifocal IOLs. Because the separate contributions of the IOL design and the refractive strategy cannot be determined from this uncontrolled series, larger prospective comparative studies are required to determine the optimal degree of mini-monovision and to compare this strategy directly with bilateral emmetropic targeting and other presbyopia-correcting IOL technologies.

## Data Availability

The datasets used and/or analyzed during the current study are available from the corresponding author on reasonable request.

## Abbreviations

IOL: Intraocular lens
EDOF: Extended depth-of-focus
UDVA: Uncorrected distance visual acuity
CDVA: Corrected distance visual acuity
UIVA: Uncorrected intermediate visual acuity
DCIVA: Distance-corrected intermediate visual acuity
UNVA: Uncorrected near visual acuity
CNVA: Corrected near visual acuity
D: Diopter
